# Temporal Changes in Incidence Rates of the Most Common Gynecological Cancers in the Female Population in Central Serbia

**DOI:** 10.3390/medicina58020306

**Published:** 2022-02-17

**Authors:** Miodrag M. Stojanovic, Natasa K. Rancic, Marija R. Andjelković Apostolović, Aleksandra M. Ignjatović, Dijana R. Stojanovic, Vesna R. Mitic Lakusic, Mirko V. Ilic

**Affiliations:** 1 Faculty of Medicine Nis, University of Nis, 18000 Nis, Serbia; drmstojanovic@gmail.com (M.M.S.); drmari@gmail.com (M.R.A.A.); drsalea@yahoo.com (A.M.I.); dijana.stojanovic@medfak.ni.ac.rs (D.R.S.); mirkoilic1971@gmail.com (M.V.I.); 2 Institute for Public Health Nis, 18000 Nis, Serbia; 3 Institute for Emergency Medical Aid, 81110 Podgorica, Montenegro; vesnamiticlakusic@gmail.com

**Keywords:** gynecological cancer, women, incidence, age, trend

## Abstract

*Background and Objectives:* There were 1,335,503 newly diagnosed cases of the most common gynecological cancers in women (cervical, uterine and ovarian cancer) worldwide in 2020. The main objective of this paper was to assess temporal changes in incidence rates of the most common gynecological cancers and to determine the age group with the greatest increase in incidence in the Serbian female population in the period 2003–2018. *Material and Methods:* Trends and annual percentage change (APC) of the incidence rate with corresponding 95% confidence intervals (CI) were calculated by Joinpoint regression analysis. The trend was considered to be significantly increasing (positive change) or decreasing (negative change) when the *p*-value was below 0.05 (*p* < 0.05). *Results:* The total number of newly registered cancer cases from 2003 to 2018 was 35,799. There was a significant increase of age standardized rate (ASR) for all cancer incidences in women from 2012 to 2018 with APC 6.9% (95% CI from 0.9 to 13.3, *p* = 0.028) and for uterine cancer during the 2014–2018 period with APC of 16.8% (95% CI: from 4.0 to 31.1, *p* = 0.014), as well as for ovarian cancer incidence in the 2012–2018 period with APC of 12.1% (95% CI: from 6.7 to 17.8, *p* < 0.001). A non-significant decrease of ASRs of incidence for cervical cancer was determined from 2003 to 2015 with APC of −0.22% (95% CI: from −3.4 to 3.1, *p* = 0.887) and a non-significant increase of ASRs incidence from 2015 to 2018 with APC of 14.21% (95% CI: from −13.3 to 50.5, *p* = 0.311). The most common gynecological cancers were present in all age groups and only ovarian cancer was registered in the youngest age group (0–4 years). Cervical cancer showed a typical increase after the age of 30, with peak incidence in women aged 40–44 and 65–69 years. The increased incidence trend regarding age for cervical cancer (y = 1.3966x + 0.3765, R^2^ = 0.3395), uterine cancer (y = 1.7963x − 5.4688, R^2^ = 0.5063) and ovarian cancer (y = 1.0791x − 0.8245, R^2^ = 0.5317) is statistically significant. *Conclusion:* Based on our presented results, a significant increase of incidence trend for the most common gynecological cancers in the Serbian female population from 2012 to 2018 was determined. There has been a significant increase in the incidence of uterine cancer from 2014 up to 2018, as well as for ovarian cancer from 2012 up to 2018, while cervical cancer showed a non-significant decrease of incidence trend from 2003 until 2015 and then a non-significant increase. In women below 20 years of age, ovarian cancer was significantly more prevalent, while cervical cancer was significantly more prevalent in the age groups 20–39 and 40–59 years. In the age group of 60–79, uterine cancer had a significantly higher incidence than the other two cancers. Measures of primary prevention, such as vaccination of children against *Human Papilloma Virus* and screening measures of secondary prevention, for the female population aged 25 to 64 years of age are needed, as well as educating females about healthy lifestyles via media and social networks to help prevent the most common gynecological cancers.

## 1. Background and Objectives

In 2020, there were 1,335,503 newly registered cases of the most common gynecological cancers in women (cervical cancer, uterine cancer and ovarian cancer) worldwide [1]. Cervical cancer (CC) had the highest incidence rate in 2020, which was 13.3/100,000; the incidence rate of uterine cancer (UC) was 8/100,000; and ovarian cancer (OC) had an incidence rate of 6.6/100,000 [1,2]. These three gynecological cancers make up 1.6% of the total number of all registered cancers of women in the world [2]. 

The highest incidence rates for all female cancers are registered in Australia at 363.0/100,000 and in 11 highly developed countries (New Zealand, Hungary, Belgium, Canada, Denmark, Ireland and the USA, The Netherlands, Norway and South Korea—around 300/100,000). The lowest incidence rates (<40/100,000) were recorded in Central America, East and Central Africa, and South and Central Asia. Most countries in Europe have high incidence rates for malignant diseases among women [3]. 

The most frequently registered gynecological cancer in women in 2020 was CC with 604,127 newly diagnosed cases, which accounted for 6.5% of all new cancer cases in women. CC was one of the three most common cancers in women in 90% of the countries in the world. The highest incidence of CC was registered in Sub-Saharan Africa, and an increase in rates was recorded in East Africa (Malawi had the highest incidence and mortality rates in the world), and Southern and Central Africa. The lowest incidence rates were in North America, with incidence rates 7 to 10 times lower in Canada (7.5/100,000) and in the United States of America (USA) (7.9/100,000), than in Australia/New Zealand and Western Asia (Saudi Arabia and Iraq) [4,5]. The incidence and mortality rates of CC in Latin America are seven times higher than in North America [6].

In the countries with well-organized population screening, the number of women suffering from CC and dying from it has decreased significantly. A significant decline in incidence and mortality has been recorded in England, Finland and Island, and this is closely related to the quality of organized screening. One of the best examples is Finland, where organized screening was introduced 45 years ago and where mortality rates decreased by 80% since that time [7]. Recently, an increased incidence has been recorded among young women aged 20–24 years and girls in Great Britain [8] and in Japan [9]. Despite still being high, incidence rates also declined in the Caribbean and Central and South America (Argentina, Chile, Costa Rica, Brazil and Colombia) during the 2000s [10]. 

Due to insufficiently effective screening in Eastern Europe [11,12] and Central Asia [13], there was a sharp increase in incidence across all ages, and an increase in premature mortality in the younger generations aged 15–44. This increase was registered in women in Sub-Saharan Africa, Ghana, Kenya, Malawi, Seychelles, Southern Africa, Uganda and Zimbabwe [14].

Uterine cancer (UC) is the most common gynecological cancer in the Western world, with more than 100,000 new patients annually in Europe alone [15,16]. The highest incidence is seen for Canada and the USA. In the USA, UC is the fourth most common malignant tumor in women. It represents the most frequently diagnosed gynecologic cancer [1] and is linked to an extremely high individual and socio-economical disease burden. According to the GLOBOCAN (accessed on 4 February 2022) the UC was the sixth most common cancer in women, and in 2020, a total of 417,000 new cases were registered. Incidence rates of this cancer vary as much as ten times worldwide and are the highest in North America, Europe, Micronesia/Polynesia, Australia and New Zealand, and lowest in Africa and South and Central Asia [1,2]. 

Known risk factors are obesity-related exposure to estrogens, older age (≥55 years), tamoxifen use, early age at menarche and late-onset menopause, while diabetes is still debated [17]. The increase in the prevalence of risk factors, primarily weight gain and physical inactivity, [18,19,20] have contributed to the increase in the incidence of this cancer in the younger generations.

The highest age-standardized incidence rates of OC in 2020 were recorded in Central and Eastern Europe with 10.7 per 100,000 [1]. OC was the seventh most common cancer worldwide and the third most common cancer in women, which represented 3.4% of all cancer cases in 2018 [3,21]. The countries that were among the ten with the highest incidence rate in 2018 were Brunei, Belarus, Poland, Ukraine, Fiji, Lithuania and Croatia. Indonesia, the United Kingdom (UK), the Czech Republic and Japan had the lowest rates—less than 10/100,000. The etiology of OC is still not well known but there have been some risk factors identified so far such as age, family history and reproductive factors, while possible risk factors include fertility drug use, hormonal substitution therapy, oral contraceptives and obesity [21]. OC is the most lethal gynecological malignancy and the five-year relative survival rate ranges from 30% to 45%, without significant improvement in recent years even though new methods in therapy have been used [22].

According to the World Health Organization (WHO), Serbia has the highest incidence of CC (24.3/100,000), compared to other republics of ex-Yugoslavia [23]. Mortality depends on incidence, and the average annual age-standardized mortality rate of 17.2 per 100,000 women in the period 1991–2010 placed Serbia among the European countries with the highest gynecological cancer mortality rates [24]. According to GLOBACAN data, the total number of new cancer cases registered among women in Serbia were 23,515 in 2020. The age-standardized rate of incidence in women compared to the world standard population was 281.3 in 100,000 women. There were 3711 newly diagnosed cases among women belonging to the three most common cancers of female genital organs. UC was present with 6.5% and was the fourth most common cancer in women, while CC was in the fifth place with a share of 5.1% [1]. In 2018, according to GLOBOCAN data, Serbia had the highest incidence rate of OC in the world—16.6/100,000 [3]. 

The main objective of the paper was to assess temporal changes in incidence rates of the most common gynecological cancers in the Serbian female population over the period of 2003–2018.

An additional objective was to determine the age group of women with the greatest increase in incidence of the most common gynecologic cancers. 

## 2. Material and Methods

This descriptive study provides data on the incidence of cancers in females from 2003 to 2018 obtained from the population-based Cancer Registry of Serbia [25]. Non-standardized, specific and standardized incidence rates per 100,000 people were calculated. We performed the analysis only for Central Serbia without the northern and southern provinces. The data from Kosovo and Metohija have not been available in the population register since 1997. Rates were standardized by the Sagy [26] direct method, and the world standard population was used as the standard. The data on the population of Serbia were obtained from the 2002 and 2011 censuses.

The code for-C53, UC-C54 and OC-C56 were used according to the 10th revision of the International Classification of Diseases [27]. We used the data from already published official publications, Cancer Incidence and Mortality in Central Serbia http://www.batut.org.rs/index.php?content=185, accessed on 25 June 2021, and Malignant tumors http://www.batut.org.rs/index.php?content=2096, accessed on 25 June 2021, from the website of the Institute of Public Health of Serbia.

### Statistical Analysis

Crude rates and age-specific and age-standardized rates (ASRs) of incidence were calculated per 100,000 inhabitants. Trends and annual percentage change (APC) of the incidence rates with corresponding 95% confidence intervals (CI) were calculated by Joinpoint regression analyses. The optimal number of Joinpoints was identified using the Monte Carlo permutation method. For the regression analyses, the Joinpoint Regression Program version 4.8.0.0. (of the USA National Cancer Institute) was used (available at http://surveillance.cancer.gov/joinpoint accessed on 25 June 2021). Trend lines were also calculated. The trend was considered to be significantly increasing (positive change) or decreasing (negative change) when the *p*-value was below 0.05 (*p* < 0.05). 

## 3. Results

The total number of newly registered cases in the observed 16-year period was 35,799. The average number of newly registered cases per year was 2200. Thus, the most common gynecologic cancers in women accounted for 1.6% of the total number of newly registered women in the structure of the incidence of cancer cases from all localizations. The largest number of newly registered women was for CC—15,667, followed by UC—11,763, with the lowest number for OC—8369. 

The annual ARS incidence rates of all registered cancers increased substantially during the observed period. A sudden jump of ARS was recorded in 2016, and the highest incidence rate of 648.7/100,000 was noticed in 2018 (Table 1).

Table 1 shows the number of newly registered cases and age-standardized incidence rates (per 100,000 people in the female population) of all cases and the three most common gynecologic cancers in the female populations of Central Serbia for the period 2003–2018.

The annual ARSs for OC ranged from 9.4 (in 2006) to 19.0/100,000 (in 2016). OC incidence increased substantially from 2010, with the highest increase in 2016 and high values both in 2017 and 2018. The lowest annual ASR of incidence for CC was recorded in 2015 (13.3/100,000) and the highest annual ASR in the whole observed period was 33.8/100,000 in 2016. The annual ASR for CC was 2.5 times higher than in 2015.

The annual ARSs for UC ranged from 12.7/100,000 (in 2003) to 25.6/100,000 (in 2017). The sudden jump of UC incidence rate was recorded in 2016 (24.8/100,000), about two times higher than the annual ASR in 2015 (Table 1). 

**Figure 1:**
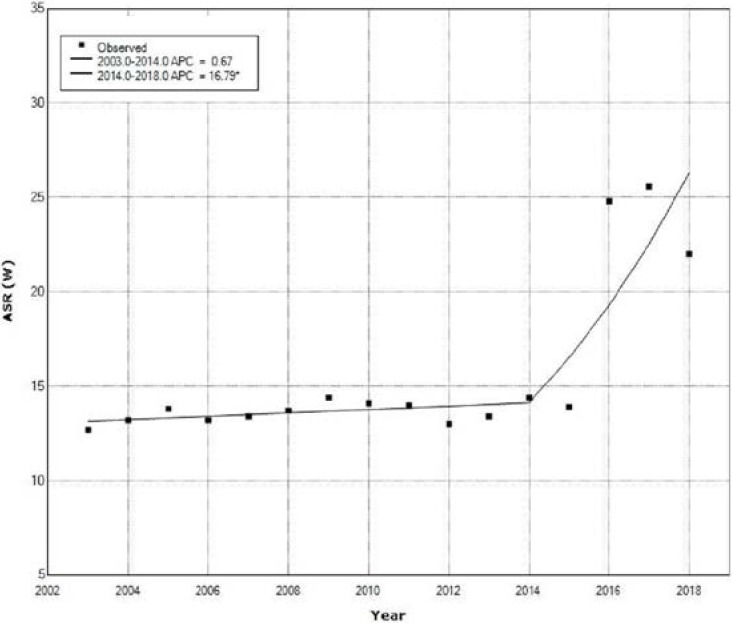
Uterine cancer trend in females of Central Serbia based on age-standardized incidence rates from 2003–2018.

Figure 1 shows the incidence trend based on age-adjusted incidence rates in females for the period of 2003–2018, using the Joinpoint (of the USA National Cancer Institute, available at http://surveillance.cancer.gov/joinpoint accessed on 25 June 2021) was used for the analysis (world standard population) of UC in Central Serbia, with annual percentage change (APC). 

A non-significant increase of ASR of incidence for UC was present in females from 2003 to 2013 with an APC of 0.7% (95% CI: from −1.8 to −3.2, *p* = 0.564). Joinpoint regression analysis showed a significant increase of ASR for UC incidence in females during the 2014–2018 period with an APC of 16.8% (95% CI: from 4.0 to 31.1, *p* = 0.014) (Figure 1). 

**Figure 2:**
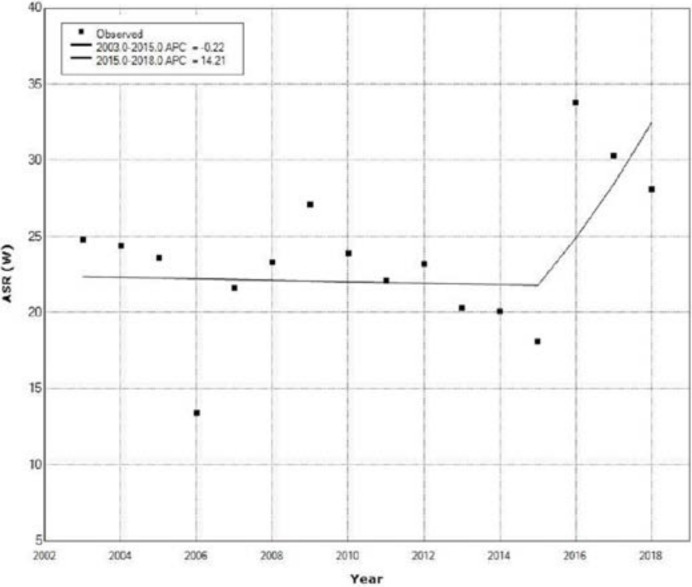
Cervical cancer trend in women of Central Serbia based on age-standardized incidence rates in the period 2003–2018.

Figure 2 shows the CC incidence trend based on age-standardized incidence rates in females from the 2003 to 2018 and results of Joinpoint analysis. 

A non-significant decrease of ASRs of incidence for CC was determined from 2003 to 2015 with an APC of −0.22% (95% CI: from −3.4 to 3.1, *p* = 0.887). Joinpoint regression analysis showed a non-significant increase of ASRs for CC incidence from 2015 to 2018 with an APC of 14.21% (95% CI: from −13.3 to 50.5, *p* = 0.311) (Figure 2).

**Figure 3:**
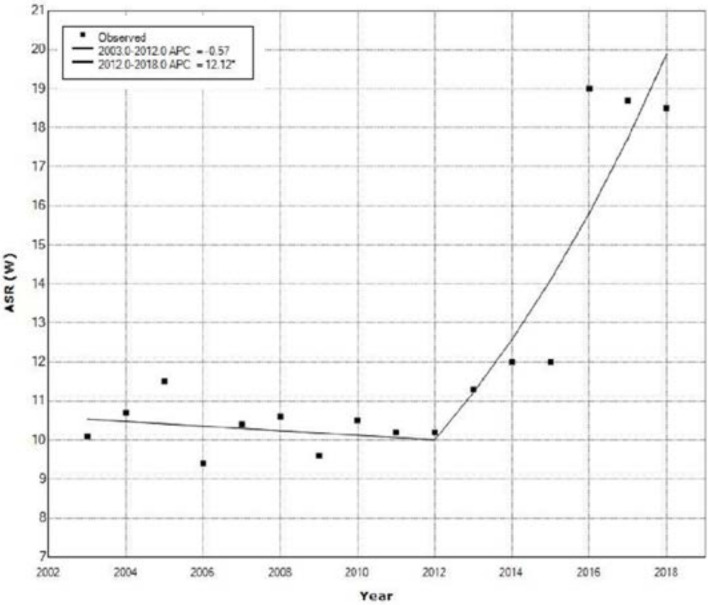
Trend of ovarian cancer in females of Central Serbia based on age-standardized incidence rates from 2003–2018.

Figure 3 shows the OC incidence trend based on age-adjusted incidence rates in females from the 2003 to 2018 and results of Joinpoint analysis.

Joinpoint regression analysis showed a significant increase of ASR for OC incidence in females during the 2012–2018 period with an APC of 12.1% (95% CI: from 6.7 to 17.8, *p* < 0.001) (Figure 3). 

**Figure 4:**
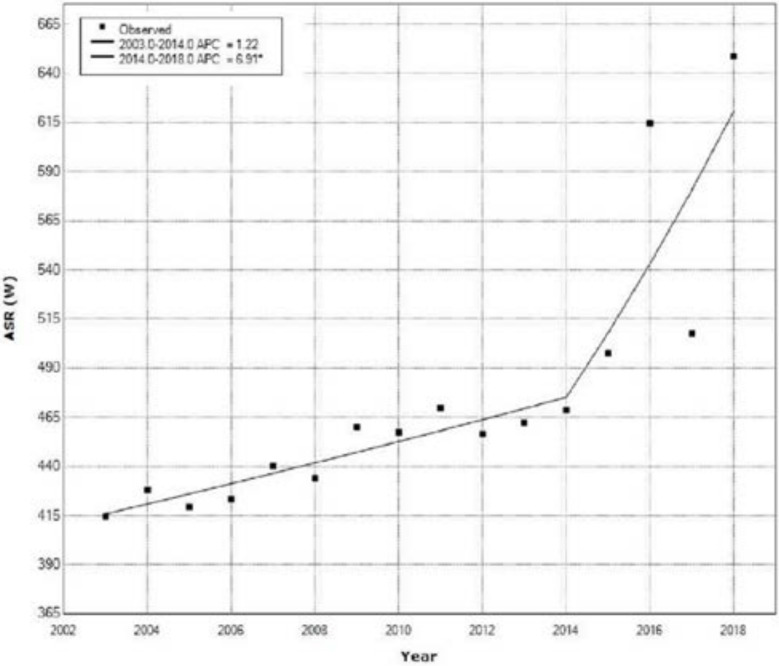
Trend of all cancers in women of Central Serbia based on age-standardized incidence rates from 2003–2018.

Figure 4 shows the incidence trend of all registered new cancer cases in females based on age-adjusted incidence rates from the 2003 to 2018 and results of Joinpoint analysis.

Joinpoint regression analysis showed a significant increase of ASR for incidence of all cancers in women from 2012 to 2018 with an APC 6.9% (95% CI: from 0.9 to 13.3, *p* = 0.028). In the period from 2003 to 2014, analysis showed an increase of the incidence trend with an APC of 1.2%, which was insignificant (Figure 4, Table 2). 

Table 2 shows the results of Joinpoint regression analysis. 

Table 3 shows the distribution of newly registered cases of cervical, uterine and ovarian cases per age group and the primary localization of cancer in the period from 2003 to 2018.

There was a significant difference between the examined groups (χ^2^ = 2324.33; *p* < 0.001). In the age group below 20, OC was significantly more common (*p* < 0.001). At the age of 20–39 (*p* < 0.001) and 40–59 (*p* < 0.001), CC was significantly more prevalent compared to the other two cancers, while in the age group 60–79, UC had a significantly higher incidence compared to the other two cancers (*p* < 0.001) (Table 3).

The total number of new CC cases in women above 60 years of age was 9300 (62.4%), and new UC cases in women younger than 60 years of age was 11764 (38.0%), more than one third. The largest number of new cancer cases in women under 60 years was for OC cases. There were 3955 (47.3%) new OC cases (Table 3).

**Figure 5:**
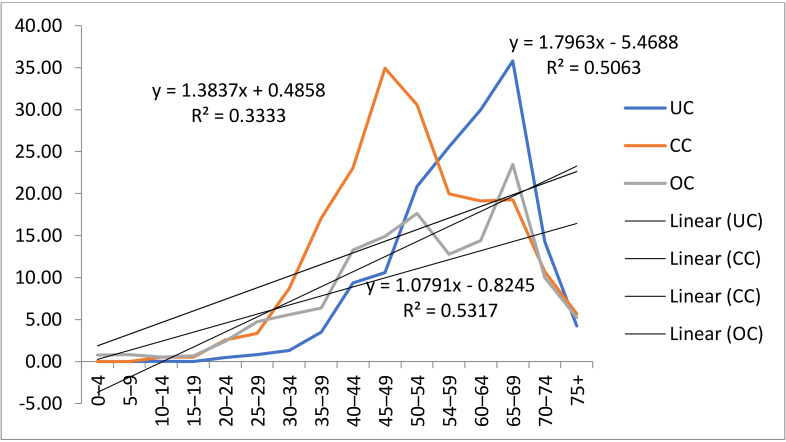
Linear trend based on age-standardized incidence rates of uterine, cervical and ovarian cancers in women from Central Serbia for the period from 2003 to 2018.

Figure 5 shows the incidence trend based on age-standardized rates of UC, CC and OC in women from Central Serbia in the period from 2003 to 2018.

The trend lines show that the incidence of all three gynecological cancers increases with age. The increased incidence trend regarding age for CC (y = 1.3966x + 0.3765, R^2^ = 0.3395), UC (y = 1.7963x − 5.4688, R^2^ = 0.5063) and OC (y = 1.0791x − 0.8245, R^2^ = 0.5317) was statistically significant (Figure 5). 

Cervical, uterine and ovarian cancers are shown in children up to the age of 15. Only OC was registered in the youngest age group (0–4 years). One new case of UC was registered in a child up to the age group of 10–14 years (Figure 5).

An increase in CC incidence is seen from the age of 20. CC incidence ASRs increased substantially, with the highest increase in the age group of 40–44 years and 65–69. The rates of OC were the highest in the age group of 45 to 54 years. UC is seen starting from the age of 20 and a rapid increase after 50 years of age has been observed. The highest rates were in the age group of 54–69 years. After 69, there was a decrease in the incidence rate of all three cancers, the lowest being in women over 75.

## 4. Discussion

We found a continuous increase in the incidence rates of all registered cancers in women in the period 2003–2018 in Central Serbia. Analysis showed a significant increase of ASRs for incidence of all cancers from 2012 to 2018. There was a significant increase in the incidence of uterine cancer from 2014 up to 2018, as well as for ovarian cancer from 2012 up to 2018, while cervical cancer showed a non-significant decrease of incidence from 2003 until 2015 and then an insignificant increase. We determined that three of the most common gynecological cancers were present in all age groups. Cervical, uterine and ovarian cancers were noticed in children up to the age of 15. Only ovarian cancer was registered in the youngest age group (0–4 years), and one new case of uterine cancer was registered in a child in the age group of 10–14 years. We also determined that cervical cancer showed a typical increase after the age of 30, with peak incidence in younger women aged 40–44 and 65–69 years of age. We also found a significant difference in the number of new cancer cases in relation to women’s age and primary localization of cancer. In women below 20 years of age, ovarian cancer was significantly more prevalent, while cervical cancer was significantly more prevalent in the age groups 20–39 and 40–59 years. Uterine cancer was the most common in the age group 60–79 years, and 51.8% of all new cases were from this age group.

The decrease of incidence of CC from 2003 until 2015 can be explained by more actions taken by the state beginning in 2006 in terms of introducing organized screening for CC, and in 2008 legal acts were also introduced [28]. The program defined all the requirements for organizing screening of CC as well as the methodology of screening. The percentage of women aged 25 to 69 who took the Papanicolaou test less than three years prior to the National Health Survey in 2019 showed an increase from 38.5% in 2006 to 57.1% in 2013 and 64.0% in 2019 [29].

In less than three years preceding the National Health Survey from 2019, screening for early detection of CC (Papanicolaou test) was carried out by two thirds of women (67.4%) aged 25 to 64 years (target population for early detection of CC) [29]. 

Mihajlović et al. (2013) found that in the period of 1999–2009 there was an increase of incidence and mortality trends for all malignant tumors in both sexes in Serbia [30]. 

In Serbia, according to the GLOBOCAN data, UC was the fourth most common cancer in 2020 [1]. The highest incidence rates for UC were registered in women in Northern America (21.2/100,000), Belarus, Samoa, Macedonia, Lithuania, Canada, Greece, Ukraine, USA, Slovakia and Croatia. Serbia occupied 11th place, with a standardized rate of less than 20/100,000 [29]. Such a high incidence of UC can be associated with an increase in the prevalence of risk factors, primarily obesity and physical inactivity, which have contributed to the increase in the incidence of this cancer in the younger generations [17,19,29].

An increase in UC incidence may be associated with a number of risk factors, including increased compounded bioidentical hormone therapy (CBHT) use, obesity and diabetes, as well as decreased use of approved estrogen–progestogen hormone therapy (HT) [17]. 

According to the findings from the National Health Survey from 2019 [29], prevalence of the well-known risk factors for UC is high. More than half of the Serbian population (57.1%) were overweight (36.3%) and obese (20.8%), based on the BMI calculation. Obesity was approximately the same in both sexes (men 21.7%, women 20.0%). The percentage of obese residents in 2019 differs slightly compared to 2013 (21.2%) but is significantly higher compared to 2006 (17.3%). In 2019, significantly more residents mostly sat or stood during work tasks in the Belgrade region (54.9%), which was more common for women (45.6%), whereas the habit of playing sports and recreation was more common in men (11.0% conduct recreational activities at least three times a week) than in women (6.9%) [29].

The incidence rates of UC have increased or stabilized since the late 1990s in many parts of the world. The interests of Japanese and Chinese scientists in UC may be explained by the increasing incidences of this malignancy in both countries [31,32]. For Japan, it is noteworthy that the numbers of affected women under 40 years of age are growing steadily, which suggests a local need to scientifically focus on fertility preservation strategies [9,31]. For Chinese women, UC is the second most common cancer of the female genital system [32].

Several studies from the UK reported increasing trends of incidence of all cancers, including UC, which remained a common malignancy in females over several decades [6,7]. The incidences were found to be slightly increased from 8% in 1984 to 10% in 2007 and were projected to be 11% in 2030, according to a study by Mistry [15]. 

CC was the fourth most common cancer in women worldwide, while in Serbia it was the fifth most common cancer in women. In Europe, the country with the highest incidence rate was Switzerland, with a rate of 75.3/100,000, followed by African countries (Malawi, Zimbabwe, Tanzania, Burundi, Uganda, Lesotho, Madagascar, etc.) with rates from 72.1/100,000 (Malawi) to 33.8/100,000 (Kenya) [11,14]. 

Kazakhstan has a high incidence of CC in women of all ages, with a crude incidence rate of 18.2 per 100,000 women [30,31]. The high incidence rates of CC and the unfavorable trend in moderately developed and the least developed countries are a consequence of the non-existence or insufficient coverage of CC screening, as well as insufficient knowledge about HPV infection [13,33].

Based on the combined data from the registries of England, Scotland, Wales and Northern Ireland, 25,033 new cases of CC were recorded in Great Britain in 2007, which is around ten times less compared to invasive forms of the disease in the country. Of the number, 95% were younger than 45 years old, and the peak incidence of CC was in the age group of 25–29 years [8,15].

According to our results, the age distribution of CC in Central Serbia from 2003 to 2018 showed a typical increase after the age of 30, with a peak incidence in women aged 40–44 and 65–69. This trend of reaching peak incidence at younger age, which has been constantly observed during the past several decades [3], indicates that CC is an age-related disease, particularly afflicting women of reproductive age [12,24].

Research has shown that *Human Papilloma Virus* (HPV) tests can be used as a basic screening tool for CC, therefore, some countries have already included it in their screening recommendations as a basic screening test in various combinations with cytological smears. The Netherlands was the first country to introduce a primary HPV test in organized screening [34].

The observed differences in CC incidence and mortality can be explained mainly by inequalities in CC screening. Numerous international studies have shown that most women do not participate in screening programs because they do not have adequate knowledge about CC, they are not aware of the frequency and severity of the disease or are unaware of the usefulness of screening in early detection of premalignant and malignant cervical lesions. As a theoretical basis for many studies, the health belief model (HBM) has been most often used to examine the behavior of women toward CC screening [24]. 

The connection between women’s knowledge and attitudes about CC and Papa testing and HPV vaccines on the one hand, and their participation in screening on the other, has been proven in a large number of studies worldwide [35]. 

In Serbia, less than one-third of women with CC are detected in the early phase of the disease, and most cases are detected when the cancer has metastasized [12,24,36,37]. The most important risk factors are chronic HPV infection [30] and smoking, which are quite common in Serbia. According to the findings from the National Health Survey, in 2000 38.1% of Serbian women were smokers. In 2019 there were less women smokers at 30.1%, and the highest percentage of smokers of tobacco products was in the age group of 45 to 54 years (41.3%). In the population of young people aged 15 to 19, every seventh (14.4%) individual stated that they consumed some tobacco products.

Differences in the incidence trend between highly developed and developing countries may be due to differences in reporting of cancer cases, changes in reproductive factors, use of oral contraceptives, substitution therapy in menopause, smoking, alcohol use, obesity and HPV vaccination. Oral contraceptives were used by about one out of ten (11.6%) women aged 20–24 and by one out of eight (13.1%) in the age group of 45–49. However, the use of menopausal hormone therapy was very low among women in Serbia—less than 1.0% [36]. 

Cervical HPV infection was detected in 19.1% of asymptomatic young women with normal cytology in Serbia [24]. The HPV vaccination is recommended but not obligatory for children at the age of nine years old and was introduced into the national immunization program in 2017 [38].

According to our findings, OC is the most common of the gynecological cancers in the female population younger than 20 years in Serbia, and nearly 50% of all new OC cases in the observed period were in females under 60 years of age. In Europe, the OC incidence is increasing in women over 70 in Denmark and Germany, in women over 65 in Finland, in women over 35 in Lithuania and Poland, but also in Thailand, Ecuador and Korea [1,22]. Incidence rates are declining in the Netherlands, Ireland, Russia, New Zealand, Singapore, the UK, Slovenia and Estonia among women aged 30 to 65, while in France as well as in Japan, this decline is seen in the age group from 30 to 70 [8]. 

The incidence trends of OC may also be partially influenced by changes in diagnostic facilities and disease classifications, particularly in high-income countries (e.g., echography, CT scan and endoscopy) (7). The increasing trends may be due to the increased prevalence of smoking, Westernized dietary patterns, obesity and the decreased prevalence of parity. Some of the possible risk factors for OC are more frequent in Serbia [39].

The increased risk of birth cohort in OC incidence was observed for most countries in Asia, Central and Eastern Europe, and Central and South America [22,33]. In the USA, despite the higher incidence rates of OC in white women (12.8/100,000) compared to black women (9.8/100,0000), black women have a poorer prognosis and shorter survival because they are more often diagnosed with OC at an advanced stage [40]. 

### Prevention and Age Strategies

Cervical cancer is considered almost completely preventable because of highly effective primary measures (HPV vaccine) and secondary measures (organizing screening, Papanicolaou tests or HPV DNK tests). In addition to chronic HPV infection, other risk factors also play an important role in development of CC. Among them, the most notorious are smoking, immunodeficiency, long-term use of oral contraceptives, promiscuous behavior and having more than two pregnancies. Family history of CC, other sexually transmitted diseases (STD’s), obesity and poor diet are also associated with CC [24,35,36,37].

Although in Serbia there is an organized CC screening program and the HPV vaccine is recommended and available for purchase, CC remains one of the most prevalent malignancies. Therefore, more public education is needed for better understanding of CC, its risk factors and ultimately better disease prevention. Such education programs are only efficient when well-planned with carefully selected target groups, such as parents, teachers and/or professors in primary and secondary schools, students, teenagers, pregnant women and women post-menopause [35]. 

## 5. Conclusions

According to the presented data, a significant increase of ASRs for all cancer incidences in the Serbian female population from 2012 to 2018 was determined. There has been a significant increase in the incidence of uterine cancer from 2014 up to 2018, as well as for ovarian cancer from 2012 up to 2018, while cervical cancer showed a non-significant decrease of incidence from 2003 until 2015 and then an insignificant increase. In women below 20 years of age, OC was significantly more prevalent, while CC was significantly more prevalent in the age groups 20–39 and 40–59 years old, and the peak incidence of CC was in the age group of 40–45 years of age. A significant increase of UC incidence from 2014 to 2018 was found in the age group of 60–79 years. It is known that primary prevention is a particularly effective way to control cancer, such as decreasing the prevalence of known risk factors (smoking, alcohol consumption, obesity and physical inactivity) in girls, young women and adults. That is why measures of primary and secondary prevention are urgent, especially for ovarian and uterine cancers. 

## Figures and Tables

**Table 1:** New cases of the three most common gynecological cancers and all cancers in females and age-standardized incidence rates in Central Serbia for the period 2003–2018.

Year	Ovarian Cancer		Cervical Cancer		Uterine Cancer		All Cancers	
	Number of New Cases	ASR * (W)	Number of New Cases	ASR (W)	Number of New Cases	ASR (W)	Number of New Cases	ASR (W)
2003	459	10.1	1004	24.8	633	12.7	11,594	414.5
2004	472	10.7	967	24.4	630	13.2	11,954	428
2005	500	11.5	948	23.6	702	13.8	11,690	419.4
2006	428	9.4	1053	13.4	646	13.2	12,835	423.3
2007	484	10.4	889	21.6	659	13.4	12,187	440.2
2008	458	10.6	945	23.3	676	13.7	11,974	434
2009	430	9.6	1104	27.1	719	14.4	12,651	460.1
2010	464	10.5	993	23.9	715	14.1	12,531	457.3
2011	482	10.2	882	22.1	695	14	12,819	469.7
2012	453	10.2	986	23.2	722	13	12,358	456.6
2013	553	11.3	863	20.3	692	13.4	12,456	462.3
2014	517	12	836	20.1	748	14.4	12,571	468.5
2015	543	12	777	18.1	702	13.9	14,582	497.6
2016	721	19	1239	33.8	973	24.8	19,115	614.6
2017	715	18.7	1124	30.3	990	25.6	19,535	507.8
2018	690	18.5	1057	28.1	861	22	22,066	648.7

* ASR (W)—age-standardized incidence rate by world standard population.

**Table 2:** Joinpoint regression models for uterine cancer, cervical cancer, ovarian cancer and all cancers in women in Central Serbia based on age-standardized incidence rates from 2003–2018.

Segment	Lower Endpoint	Upper Endpoint	APC	Lower CI	Upper CI	Test Statistic (t)	Prob > |t|
**Uterine Cancer**							
**Count**							
ASR W*							
1	2003	2014	0.7	−1.8	3.2	0.6	0.564
2	2014	2018	16.8 *	4.0	31.1	2.9	0.014
**Cervical Cancer**							
**Count**							
**ASR W**							
1	2003	2015	−0.2	−3.4	3.1	−0.1	0.887
2	2015	2018	14.2	−13.3	50.5	1.1	0.311
**Ovarian Cancer**							
**Count**							
**ASR**							
1	2003	2012	−0.6	−3.2	2.1	−0.5	0.645
2	2012	2018	12.1 *	6.7	17.8	5.1	**<0.001**
**All Cancers**							
**Count**							
**ASR W**							
1	2003	2014	1.2	−0.0	2.5	2.1	0.055
2	2014	2018	6.9 *	0.9	13.3	2.5	0.028

ASR W *—Age-standardized rate according to the world standard population.

**Table 3:** Distribution of the most common gynecological cancers in women from Central Serbia per age and cancer site from 2003 to 2018.

Cancer Site	Age Groups									
	0–19		20–39		40–59		60–79		Total	
	*n*	%	*n*	%	*n*	%	*n*	%	*n*	%
CC *	4	0.03	2027	12.94	7749	49.46	5887	37.58	15,667	100
UC ^§^	1	0.01	255	1.63	4200	26.81	7307	46.64	11,763	100
OC **	50	0.32	705	4.50	3200	20.43	4414	28.17	8369	100

* Cervical cancer; ^§^ Uterine cancer; ** Ovarial cancer.

## Data Availability

The data presented in this study are available on request from the corresponding author.

## Abbreviations

GLOBOCAN	Global Cancer Observatory
CC	cervical cancer
UC	uterine cancer
OC	ovarian cancer
ASR	age-standardized rate
APC	annual percentage change
CI	confidence interval
CBHT	compounded bioidentical hormone therapy
HT	hormone therapy
